# Axillary Aneurism—Deviation of the Subclavian Artery—Recovery

**Published:** 1849-12

**Authors:** 


*Axillary Aneurism—Deviation of the Subclavian Artery—Recovery.* By Joshua B. Flint, M. D., etc., of Louisville Ky.

On the 17th of May last, Mr. G., a healthy, athletic man of five-and-twenty, living about ten miles from Louisville, was stabbed in the left shoulder. The knife entered just in front of the articulation, penetrated the axilla and wounded the artery; the hemorrhage was profuse and prostrating. Syncope coming in aid of means used by the bystanders, the bleeding was stanched until the arrival of a neighboring physician, under whose judicious and persevering efforts it was permanently arrested about fourteen hours after the injury—in the meantime, and for some hours longer, the patient being motionless and unconscious from exhaustion.

A few days after leaving his bed, he noticed a swelling in the arm-pit, and finding, after observing it a while, that it was enlarging, and interfering with the movements of the limb, he exhibited it to his physician. Dr. Foss perceived at once, the nature of the swelling, endeavored to explain to the patient its character and tendencies, and advised him to come to the city for further advice and such surgical aid as his case required.

He presented himself to me about the middle of June. In the left axilla was a pulsating tumor, exhibiting all the evidences and characteristics of aneurism. It filled the axillary cavity, the lower portion, indeed, projecting somewhat beyond the true margin of this cavity. The tumor appeared to be furnished with a very regular, well-defined cyst; the shoulder was crowded considerably upward, and the arm thrown off from the trunk at a large angle.

I repeated to the patient what his physician had told him of the nature of his disease, described to him as well as I could, the process most likely to relieve him, and advised him, as he could not then remain in the city, to return without any avoidable delay, and have the operation performed as soon as, by repose and suitable regimen, he should appear to be well prepared for it.

Arrangements were accordingly made for operating, on the 21st of June, in the presence of a number of medical gentlemen, more or less conversant with surgical proceedings. The patient was placed upon a table, with the head and shoulders so disposed as to expose as much as possible the parts to be involved in the operation. An incision, two inches and a half in length, was carried across the base of the supra-clavicular triangle, about a quarter of an inch from the bone, and this incision was joined at its inner extremity, by another about an inch and a half in length, running along the outer edge of the sterno-mastoid muscle. The flap of integuments being raised, and a similar section of the superficial fascia and platysma drawn aside, the subjacent textures were divided by the point of the knife, or pushed aside by its handle, until the usual situation of the subclavian artery between the scaleni, was attained. The edge of the outer muscle being followed down to the tubercle of the rib, the vessel was sought for by these landmarks, in its usual course. But it was sought in vain—it was not there. In its place, exactly, was the lower cord of the cervical plexus of nerves. Having satisfied myself that the vessel was not *in situ*, and given a moment to the emotions of surprise and disappointment the discovery occasioned, the intruding nerve being drawn to the outer side of the wound, I proceeded to explore its recesses for the errant artery. Guided by the pulsations, which had been felt obscurely during the whole progress of the operation, I pushed my way with the finger and handle of the scalpel, under the anterior scalenus muscle, until the extremity of the finger could be placed upon the naked vessel, about an inch and a half from the outer margin of the muscle, in a direction npward and inward. Only a small portion of the tube, such as could be covered by the end of the fore-finger, was, or could be uncovered. On every side it seemed to be enveloped by muscular masses that would not yield to any force which it was thought prudent to make in attempting to isolate the artery.

In order to facilitate further manifestations in the depths of the wound, its orifice was now enlarged by a section of the outer margin of the sterno-mastoid muscle, and a division of some fibres of the trapezius. The essential difficulty, however, still remained; consisting of the depth and obliquity of the passage to the vessel, and the closeness with which the surrounding textures embraced it. After much fruitless effort to isolate the artery sufficiently to justify a hope of being able to pass a ligature around it by means of any instrument known or imagined, I reluctantly abandoned the attempt; determined, after the patient should have recovered from the effects of the present proceeding, to give him the chances of recovery offered by a ligature on the artery beyond the aneurismal tumor. In my reflections on his case, I had at one time contemplated this operation as the primary undertaking in his behalf, believing that the artery was wounded beyond the exit of the great axillary branches, and that a ligature might be placed between the disease and any considerable muscular branch, so as to secure the most material condition of success in this method of treatment. The wound was cleansed and drawn together by suture and adhesive plaster. The patient was placed in bed, manifesting a good deal of nervous exhaustion, but suffering not at all from loss of blood, only one small vessel having been divided which required a ligature. The wound healed kindly, with very little suppuration, and in two days the patient was on his feet again, as well as he was before the operation; but, as we then supposed, no better. After a few days, however, I received a message from him, explaining why he had not come to town, according to our arrangement, by the information that he was recovering.

About three weeks after the operation, he called on me, and I was agreeably surprised to notice the improvement in his condition. The axillary tumor was very much diminished, and not the feeblest pulsation in it could be detected.

Another examination made this day, September 1st, confirms the favorable conclusions of the previous one, and leaves no doubt of a complete cure. The elastic swelling that once filled the axilla, is reduced to a solid tumor of the size of a pigeon's egg, and the shoulder and arm have almost resumed their natural position and movements. The pulsation in the artery below the point of injury, is only a little less strong than that in the other side, and above the clavicle the movements of the subclavian are distinctly felt through the indurated textures constituting the cicatrix of the secondary wound.

There are two points of great interest in this case; and I have related it with more particularity than were otherwise desirable, in order to supply the materials for such readers as choose to do so, to contemplate them critically.

1.—*The anomalous position of the subclavian.*—Exactly what are the abnormal relations which the artery here sustains, can only be known from a dissection of the parts, and, happily for the patient, his present condition forbids us to expect that this satisfaction will be realized by any living anatomist.

My own impression and belief, however, is, that after proceeding rather higher than usual in its vertical course, the artery, in becoming horizontal, turns under the posterior scalenus muscle, instead of taking the usual route between it and its anterior fellow. Confirmatory of this belief, is my recollection, that in the primary examinations of the case, it was noticed that the pressure over the clavicle, which most effectually arrested pulsation in the tumor, was directed rather backwards than downwards—against the spine rather than against the rib.

Irregularities in the origin of the left subclavian are not uncommon; and in its middle portion deviations *forwards,* such as accompanying the vein, exchanging places with the vein, penetrating the anterior scalenus muscle, etc., have repeatedly been noticed. Dr. Warren, Jr., of Boston, in an operation upon the subclavian, not long since reported, found a deviation in this portion of the vessel *forwards,* if I understand the account—the artery being thrown out of its place by some distortion of the skeleton. But I have not been able to find in any anatomical description or delineation of the blood vessels or their anomalies, any notice of a deviation of the subclavian *backwards*, like that which is presumed to exist in the present case.

Although the discovery of such an anomaly may never have rewarded the curiosity of the *anatomist,* I apprehend that more than once the *surgeon* has encountered it, with somewhat more serious emotions than those of mere curiosity.

The first recorded attempt to tie the subclavian was rendered unsuccessful in the hands of the late Sir A, Cooper, by embarrassments which I conceive were due to an unusual distribution of the artery, somewhat like that we are now considering. The accounts of this operation, given respectively by M. Velpeau, and Dr. Mott, who was in London at the time, and an eye witness of the interesting undertaking, vary so much, that it is not easy to understand what was the essential difficulty in the proceeding. According to Dr. M., Sir Astley complained of the depth of the wound; and, moreover, expressed his fears, that in attempting to uncover the artery, he had wounded the thoracic duct, and thus hastened the death of the patient, which took place six days afterwards. If there were any grounds for this apprehension, the operator must have been carried, in pursuit of the artery, very far within and behind the place where he expected to find and tie it.

A similar failure afterwards happened to Professor Lallemand, in attempting to tie the subclavian for the purpose of arresting a hemorrhage in the axilla. Here the embarrassment could not have occurred from the presence of a tumor, pressing up the shoulder and interfering with the manipulations of the surgeon, as is sometimes the case in operations for aneurisms, but can hardly be attributed to any cause except an unusual remoteness of the vessel.

If we could discover beforehand, such a deviation as I have supposed to exist in the present instance, it would not be wise to attempt to seize the artery through a wound whose primary incisions were made after the usual method of operating without the scaleni; for I deem it out of the question to succeed under such circumstances. We should do better to commence on the inside of the sterno-mastoid, the primary incision being laid much as we should shape it for finding the primitive carotid trunk.

2.—*The Recovery.*—To what is it due? We know that spontaneous cures of aneurism occasionally take place; and it is an ungracious thing to dispute about the credit of a cure, with that great physician called nature, to whom all practitioners of the healing art are under such infinite obligations. But the disease was progressing until the time of the operation, and we know of nothing that took place afterwards, either of a constitutional or local character, calculated to exercise a favorable influence upon it. The patient escaped from all professional appliances and restraints immediately after the operation, and resumed, as he was able, his usual habits of life. He presently found that the movements of the arm were executed with more freedom, and this observation led him to notice the diminution of the aneurismal tumor. Under all the circumstances, it can hardly be doubted that art, although thwarted in her plan of relief, may fairly take the credit of contributing largely towards the gratifying result.

Under the light thrown upon this department of surgery, during the last few years, in the successful treatment of aneurism by partial compression of the artery involved, we can understand in what manner and degree the contributions of art became efficacious in the present instance. All that is necessary for the consolidation and subsequent removal of an aneurismal tumor is, that the current of blood should be sufficiently retarded in the affected artery, to favor the deposition and permanence of febrine in the morbid cyst. The vessel, in the present case, was subjected to a considerable degree of pressure and friction over the small portion of it that was exposed, in attempts to separate it from neighboring parts—enough of pressure and friction to excite reaction, which propagated to the inner coat, might have determined swelling of the texture or deposition of lymph on its inner surface, sufficient to diminish the caliber of the vessel very materially, and to retard proportionately the current of blood from that point onward.

The inner coat of the arteries is remarkably prone to take an action that results in plastic effusion. We are now well assured that the violence which the experiments of Jones taught us it was necessary to inflict upon this texture, to secure obliteration of the vessel, is quite unnecessary, and that this purpose may be effected by simply bringing the inner parieties of the tube into contact, slightly irritating this surface by acupuncture, or even by irritation transmitted to this membrane from strains and bruises inflicted on the outer coats.

Moreover, it is not unreasonable to suppose that, even if these intrinsic processes were not set up in the denuded artery, by the violence to which it was subjected in the operation, sufficient diminution of its caliber to determine the same curative results, might be occasioned through the pressure exercised upon it from without, by the contraction of surrounding textures in the subsequent process of cicatrization. We do not often, it is true, notice such an occurrence in the large blood vessels, but when nervous trunks get engaged in cicatrices, the pressure exercised upon them is often sufficient to produce the most serious consequences.

By either of the two modes of contraction, however, the force of the circulation in the vessel would be broken at the contracted point, a kind of eddy produced in the portion immediately beyond it, and the most favorable conditions secured for the curative deposits to be made in the tumor.

If this interpretation of the phenomena of our case be correct, while we are indebted to the improved surgery of our Dublin brethren, for the establishment of those principles on which it depends, the most important phenomenon of the case itself—the recovery—with the reciprocity characteristic of true science, reflects additional confirmation on the principles that explain it, and affords a beautiful and encouraging illustration of their practical value.—*Western Lancet.*

